# Racial, Ethnic, and Place-of-Birth Disparities in Body Types: Association to Glycated Hemoglobin in Older Adults

**DOI:** 10.1093/geroni/igaa057.717

**Published:** 2020-12-16

**Authors:** Queendaleen Chukwurah

**Affiliations:** Tulane University, New Orleans, Louisiana, United States

## Abstract

Obesity, a public health concern for older adults, contributes to abnormal glycated hemoglobin (HbA1c). We examine place of birth disparities in the prevalence rate of body types and their association to HbA1c using the National Health Nutrition and Examination Survey (NHANES) III. Body mass index /waist circumference (WC) cut off values were used to create six body types: normal weight with normal WC, overweight with normal WC, obese with normal WC, normal weight with high WC, overweight with high WC, and obese with high WC. Abnormal HbA1c was defined as HbA1c >5.7%. Weighted multivariable logistic regression adjusted for age, gender, education, and poverty-income-ratio was run. The sample population included 4,584 participants aged 50 years and older identifying as non-Hispanic whites (NHW), US-born Hispanics (USB-H), and foreign-born Hispanics (FB-H). The mean (SD) sample age was 63.9 (0.3). USB-H had the highest proportion of obese with high WC (35.6%,p<0.0001) compared to NHW (26.6%) and FB-H (22.2%). USB-H (aOR 1.97 95% CI 1.45,2.68) and FB-H (aOR 1.51 95% CI 1.10,2.06) had higher odds of abnormal HbA1c compared to NHW. Overweight with high WC (aOR 1.47 95% CI 1.11-1.93) and obese with high WC (aOR 2.11, 95% CI 1.60-2.79) had a high likelihood of abnormal HbA1c compared to normal weight with normal WC. Further adjustment for co-morbid conditions yielded a significantly improved fitting model (Maximum-rescaled R-square (MRRS) =0.1997,p<0.0001) compared to that further adjusted for health-related behaviors (MRRS=0.082,p<0.0001). The knowledge of these associations in an at-risk sub-population is insightful for clinical assessments and preventive interventions.

